# Parabens as Agents for Improving Crocetin Esters’ Shelf-Life in Aqueous Saffron Extracts

**DOI:** 10.3390/molecules14031160

**Published:** 2009-03-16

**Authors:** Luana Maggi, Manuel Carmona, Amaya Zalacain, Magdalena Martínez Tomé, María Antonia Murcia, Gonzalo Luis Alonso

**Affiliations:** 1 Cátedra de Química Agrícola. E.T.S.I. Agrónomos, Universidad de Castilla-La Mancha, Campus Universitario, 02071 Albacete, Spain; E-mail: Luana.Maggi@uclm.es (L.M.), Manuel.Carmona@uclm.es (M.C.), Amaya.Zalacain@uclm.es (A.Z.); 2 Área de Nutrición y Bromatología. Facultad de Veterinaria y CYTA. Universidad de Murcia. Campus de Espinardo. Espinardo 30071. Murcia. Spain; E-mail: mmtome@um.es (M-M.T.), mamurcia@um.es (A.M.)

**Keywords:** Crocetin esters, Picrocrocin, Parabens, Saffron (*Crocus sativus* L.), Aqueous extract.

## Abstract

The effect of parabens on the shelf-life of crocetin esters and picrocrocin in aqueous saffron solutions was studied. Degradation of saffron crocetin esters fits a first-order kinetics model, and the results indicated that the crocetin (*β*-d-glucosyl)-(*β*-d-gentiobiosyl) esters were more stable than the crocetin di-(*β*-d-gentiobiosyl) esters regardless of whether *trans* and *cis* isomers were considered. Under all tested conditions both parabens gave good results, especially propyl paraben that showed a greater influence on the degradation rate constant, except for *cis*-crocetin di-(*β*-d-gentiobiosyl) ester and *cis*-crocetin (*β*-d-glucosyl)-(*β*-d-gentiobiosyl) ester. In presence of propyl paraben (200 mg/L), the half-life periods of* trans*-crocetin di-(*β*-d-gentiobiosyl) ester improved considerably, up to four-fold. Special attention has been paid to the effect of propyl paraben on 46 saffrons with different crocetin ester contents. No differences were observed in terms of picrocrocin. By analysis of variance, it is noteworthy that there were differences between the mean content of crocetin esters for all analysed saffron, except for* trans*-crocetin (*β*-d-glucosyl)-(*β*-d-gentiobiosyl) ester.

## 1. Introduction

When the carotenoids reduce their size, eliminating terminal groups from the molecule, they are known as apocarotenoids, the group to which the saffron pigments belong to. Derivatives of an apocarotenoid, called crocetin (C_44_H_64_O_24_, 8,8´-diapo-Ψ,Ψ´-carotenedioic acid), in which it is esterified with one or two glucose, gentibiose or neapolitanose sugar moieties, are present in *Crocus sativus* L. stigmas and *Gardenia jasminoides* Ellis fruit and are commonly known as crocetin esters [1]. These compounds are known for their colouring properties, owing to their peculiar water soluble behaviour, in contrast to most families of carotenoids. It has been demonstrated that the colouring strength as well as the visual colour of saffron, depends on the dehydration process used to produce saffron spice [2]. The crocetin esters deteriorate quickly in aqueous extracts and several kinetic studies have shown that colour degradation follows first-order kinetics; it is sensitive to exposure to light, thermal treatment, and acidic environments, as well as to the presence of additives [3,4,5,6,7,8,9]. It would be very interesting to lengthen the life of saffron aqueous extracts to avoid the fast oxidation of crocetin esters. 

Picrocrocin (C_16_H_26_O_7_,4-(β-D-glucopyranosyloxy)-2,6,6-trimethyl-1-cyclohexene-1-carboxaldehyde) is considered as being mainly responsible for the bitter taste of saffron, together with other related compounds and kaempferols [10]. Picrocrocin is converted to safranal, the main contributor to saffron aroma, either by an enzymatic/dehydration process or directly by thermal degradation. Unlike crocetin esters, only a few studies have dealt with picrocrocin degradation, and these have emphasized its high stability [3,11]. 

Compounds belonging to the paraben family, specifically methyl paraben (MP) and propyl paraben (PP), are commonly added to foods, drinks, medicines and cosmetics as antimicrobial preservatives due to their relatively non-irritating, non-sensitizing and low toxicity characteristics [12,13]. Moreover, parabens occur naturally in foods. Methyl paraben has been reported as a constituent of cloudberry, yellow passion fruit juice, white wine, botrytised wine, and Bourbon vanilla, and recently, propyl paraben has been detected in the aerial part of the plant *Stocksia brahuica* (Family: Sapindaceae) [14]. Several authors reported methods for determination of parabens in cosmetics, food products and pharmaceuticals [14,15,16,17,18,19]. For example, degradation kinetics of methyl and propyl paraben in aqueous solution are well described in literature, being in both cases first-order degradation kinetics [20]. 

For this reason, the main objective of this work was to study the effect of parabens on the life prolongation of overall, *trans*, *cis* and individual crocetin esters and picrocrocin in aqueous saffron solutions to reduce their degradation in order to know the real colour composition of saffron extract; and to make faster and cost-effective the determination of crocetin esters when a great number of samples arrive in the laboratory in a short period of time. The effect of propyl paraben on saffrons with different content of crocetin esters was also studied.

## 2. Results and Discussion

**Figure 1:**
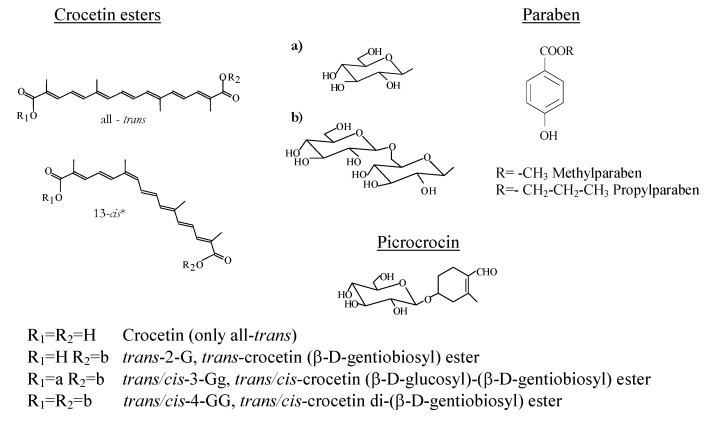
Structure of crocetin esters, picrocrocin and parabens. In the case of crocetin esters with *cis*-configuration, the position of the substitutes R_1_ and R_2_ could not be exactly determined in relation to the C13-14 bond.

Quality parameters of a control saffron sample were evaluated according to ISO 3632 (2003) [21]. Results indicated that the sample used belonged to category I: moisture and volatile content, 9.3%; colouring strength (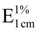 440 nm), 291; 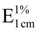 257 nm, 94; 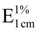 330 nm, 30. Table 1 reports the overall and individual crocetin ester composition of the control sample, expressed as percentage on a dry basis, their retention times and also their kinetic parameters as rate constants (k), determination coefficients (R^2^) and half-life periods (t_1/2_). It is noteworthy that all rate constants were negative, as it was degradation, but they were expressed in absolute value. It is necessary to point out that the *trans*-crocetin di-(*β*-d-gentiobiosyl) ester (*trans*-4-GG; Figure 1) represents almost the 60% of the total crocetin ester content present in the aqueous extract and the first two (*trans*-4-GG and *trans*-3-Gg) comprise over 80% of the total esters. The half-life period of total crocetin esters was 47 hours, while for *trans* and *cis* it was 51 and 39 hours, respectively, indicating a strong effect due to the* trans* isomer content. The results pointed out that the 3-Gg crocetin esters were more stable than the 4-GG regardless of the isomer (*trans* or *cis*) considered. 

**Table 1:** Total and individual crocetin esters composition, retention time (t_R_), rate constants (k), determination coefficients (R^2^) and half-life periods (t_1/2_) of each crocetin ester in saffron aqueous extract of the control sample.

Compound	Mean content^ab^±SD (g/100g)	% content^a^ ± SD	t_R_ (min)	(*k*±SD)*10^3^ (h^-1^)	R^2^	t_1/2_ (h)
Total crocetin esters	31.15±0.05	100.00±0.01		14.6	0.995	47
Total *trans*	28.21±0.08	93.09±0.22		13.7	0.995	51
Total *cis*	2.94±0.17	6.91±0.32		17.7	0.946	39
Trans-4-GG	18.72±0.38	58.61±1.19	10.3	29.3	0.998	24
Trans-3-Gg	6.75±0.39	25.34±1.45	10.8	11.9	0.912	58
Trans-2–G	0.87±0.12	4.08±0.56	11.5	41.8	0.972	17
Cis-4-GG	1.97±0.32	4.39±0.71	12.0	18.8	0.991	37
Cis-3-Gg	0.82±0.10	2.20±0.28	12.7	11.4	0.974	61

^a^ Values are the means of trials performed in triplicate; ^b^ g of compound/100g of saffron, dry basis.

The trials carried out at 25 ºC showed that the crocetin esters follow a first-order kinetic model in aqueous extracts. Also, with respect to the rate constants (k) and half-life periods (t_1/2_) of the major crocetin esters, the data obtained at 25 ºC were intermediate values with regard to those reported by Sanchez *et al*. and measured between 5 and 30 ºC [9].

As it can been observed in Table 1, the degradation of crocetin esters is important, but these compounds need some time for extraction. According to ISO 3632 (2003) [21] to standardize saffron quality analysis, the extraction time is set to 1 hour, but this is evidently not enough time to completely extract all the crocetin esters as the vegetable material still has an intense red colour. Other authors have reported more exhaustive extraction times, up to 24 hours [3,7], so some stabilizers such as the parabens maybe useful for addition to the aqueous saffron samples for the purpose of improving the analysis time. As well, during saffron post-harvesting period many samples have to be analysed in short period of time. Table 2 shows the kinetic parameters for *trans* and *cis* crocetin esters in saffron aqueous extracts in presence of different concentrations of methyl paraben and propyl paraben, respectively. In most cases studied the degradation of crocetin esters adjusted to a first-order kinetics model although this was not always possible since their content was constant. As has been observed, when increasing the concentration of parabens the *k* values showed a marked decrease in comparison with the data obtained for control sample. For *trans*-2-G, the degradation of two esters ceases, with their content remaining constant in presence of 100 mg/L of MP or PP and also for higher concentrations. At 150 and 200 mg/L of the two parabens *cis*-3-Gg remained at a constant level, showing the positive effect of their addition to this crocetin ester. In all tested conditions both parabens gave good results, especially propyl paraben, which showed a greater influence on the degradation rate constant. The only exceptions were *cis*-4-GG at all concentrations of methyl paraben and *cis*-3-Gg at 50 and 100 mg/L, where the degradation rate constants were higher than for propyl paraben. In the presence of MP *cis*-isomers had lowest *k* and therefore they degraded more slowly. With respect to the 3-Gg and 4-GG crocetin esters, also in presence of parabens, it was established that 3-Gg had a longer half-life period, regardless of the isomer considered (*trans* or *cis*). 

**Table 2:** Degradation rate constant (k), determination coefficient (R^2^) and half-life period (t_1/2_) of overall, *trans*, *cis* and individual crocetin esters in saffron aqueous extract in presence of methylparaben and propylparaben, respectively, at 25 ºC.

Crocetin esters	Methylparaben											
	50 mg/L			100 mg/L			150 mg/L			200 mg/L		
	(K±SD)*10^3^ (h^-1^)	R^2^	t_1/2_ (h)	(K±SD)*10^3^ (h^-1^)	R^2^	t_1/2_ (h)	(K±SD)* 10^3^ (h^-1^)	R^2^	t_1/2_ (h)	(K±SD)*10^3^ (h^-1^)	R^2^	t_1/2_ (h)
Total crocetin esters	17.6	0.989	39	14.8	0.966	47	9.4	0.974	74	8.8	0.972	79
Total *trans*	18.2	0.987	38	15.5	0.964	45	9.2	0.977	75	9.2	0.971	75
Total *cis*	6.8	0.931	101	6.4	0.930	108	4.1	0.939	169	2.6	0.840	267
Trans-4-GG	24.3	0.996	29	20.4	0.920	34	9.6	0.999	72	8.2	0.940	85
Trans-3-Gg	10.1	0.966	69	10.0	0.950	69	9.7	0.903	71	9.4	0.923	74
Trans-2-G	26.4	0.961	26	*			*			*		
Cis-4-GG	14.9	0.917	47	9.7	0.981	71	6.6	0.938	105	5.0	0.915	139
Cis-3-Gg	9.2	0.947	75	4.6	0.983	151	*			*		
	Propylparaben											
	50 mg/L			100 mg/L			150 mg/L			200 mg/L		
Total crocetin esters	14.4	0.995	48	13.1	0.983	54	11.8	0.958	83	7.5	0.988	92
Total *trans*	14.2	0.998	49	12.8	0.961	52	9.6	0.971	80	7.8	0.978	86
Total *cis*	8.0	0.940	94	7.3	0.989	99	*			*		
Trans-4-GG	14.8	0.998	47	14.3	0.990	48	8.5	0.982	82	7.4	0.981	94
Trans-3-Gg	11.7	0.980	59	4.9	0.917	141	4.3	0.944	161	4.3	0.971	161
Trans-2-G	16.2	0.930	43	15.2	0.916	46	*			*		
Cis-4-GG	16.0	0.996	43	11.4	0.922	61	8.4	0.999	83	8.2	0.923	85
Cis-3-Gg	9.9	0.946	70	9.0	0.938	77	*			*		

* First-order kinetics was not followed, crocetin ester content was constant

Since *trans*-4-GG is approximately 60% of total content, its behaviour is dominant in comparison with the behaviour of other crocetin esters. Upon increasing the concentration of propyl paraben up to 200 mg/L, the effect on *trans*-4-GG improved the half-life periods considerably, going from the 24 hours obtained at 25 ºC without the addition of paraben to 94 h after adding 200 mg/L of propyl paraben, a four-fold improvement. Moreover, the degradation rate constant decreased to 0.0074 h^-1^, so it indicates a higher stability of this compound and also a slower degradation. In the conditions used to carry out these trials, the addition of PP gave better results than MP, showing a positive effect especially on the *trans*-crocetin esters that represent the almost total content. 

The effect of two parabens on picrocrocin was then studied. In both cases, it was observed that picrocrocin follows a second-order kinetic model according to Alonso *et al*. [5]. It therefore seems that the addition of paraben did not influence its degradation since its half-life period was very long. According to Morteza *et al*. [22], the rate of methyl paraben decomposition was higher than propyl paraben.

### 2.1. Stability of saffron from different geographical zones

**Figure 2:**
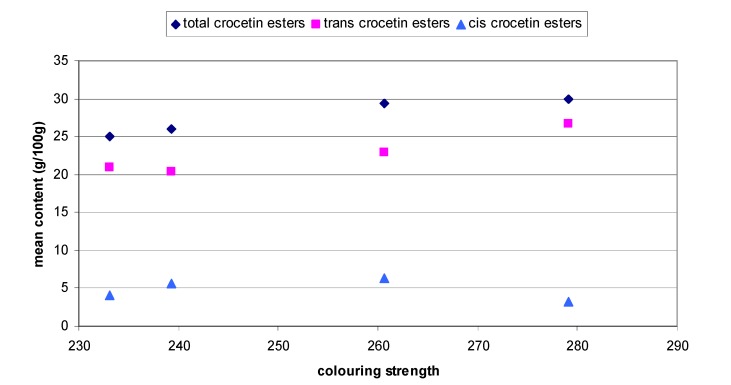
Correlation between colouring strength and mean content of total, *trans* and *cis* crocetin esters in samples from Greece, Italy, Spain and Iran.

Next the influence of propyl paraben (200 mg/L) on the degradation of saffron with different content of crocetin esters was valued on 46 saffron samples coming from four different countries (Table 3). Data indicated that these samples all belonged to category I according to ISO 3632 (2003) [21]. The saffrons coming from Greece, Italy, Spain and Iran had different colouring strength, thus showing a different content of *trans* and *cis* crocetin esters. The correlation between colouring strength and mean content of total, *trans* and *cis* crocetin esters is shown in Figure 2. As can be observed, the values of colouring strength seem to be directly proportional to the content of *trans* crocetin esters. For Greece, Italy, Spain and Iran the first two in percentage content are *trans*-isomers, representing more than 70%. For the four countries, the total content of *trans*-isomers varies in a range between 83 and 91%, so the kinetic behaviour of these saffrons will follow the trend imposed by the predominant crocetin esters. By analysis of variance (ANOVA) it is noteworthy that there were differences between the saffrons coming from Greece and Spain. This can be explained by the different production processes. Olive oil was added to Italian saffron to improve storage. Iranian saffron was processed in a different way, when compared to Greece, Spain and Italy. With regard to the first two, the content of *trans*-4-GG is different for the four countries, while *trans*-3-Gg content was not significantly different between Greece and Italy, nor between Greece and Spain, unlike Iran. Thus, the greater advantages deriving from the addition of propyl paraben will be observed in saffrons with a relative higher content of *trans*-crocetin esters. Considering the data obtained, Italian and Iranian saffrons have the higher content of *trans*-isomers, so the positive effect of PP should be greater on them. In the conditions tested, PP is able to prolong the life of crocetin esters in saffron aqueous extracts and allows for analysis of compounds with a fast degradation and also a better use of the equipments as the autosamplers. Furthermore, the addition of PP in aqueous solution could be extended to other products.

**Table 3:** Quality characteristics and content of each crocetin ester in saffron aqueous extracts coming from Greece, Italy, Spain and Iran, stabilized by means of propylparaben addition (200 mg/L).

Country	**Greece (9)**			**Italy (11)**			**Spain (14)**			**Iran (12)**		
Moisture & volatile content % ± SD	8.51±0.69			8.78±0.43			6.59±1.24			7.29±0.57		
Colouring strength ± SD	239.30±9.87			279.14±12.15			260.63±20.39			233.11±7.86		
*Crocetin Esters*	Mean content (g/100g)	(Content± SD)%	Δ mean content*±SD	Mean content (g/100g)	(Content± SD)%	Δ mean content*±SD	Mean content (g/100g)	(Content± SD)%	Δ mean content*±SD	Mean content (g/100g)	(Content± SD)%	Δ mean content*±SD
*Total*	25.94b	100.00±0.01	1.08±0.58	29.88c	100.02±0.03	1.42±1.09	29.31c	100.00±0.01	-1.94±0.19	24.99a	100.00±0.01	1.55±0.37
*Total trans*	20.37a	83.86a±0.93	0.99±0.12	26.67c	91.53c±0.65	1.45±1.07	22.98b	85.26a±1.45	-0.70±0.27	20.94a	88.09b±1.97	1.22±0.34
*Total cis*	5.57b	16.14c±0.93	0.96±0.34	3.21a	8.41a±0.65	0.73±0.41	6.33b	14.74c±1.45	1.52±0.72	4.05a	11.91b±1.97	1.60±0.46
*Trans*-4-GG	11.77a	44.79a±2.57	2.29±0.59	17.79c	58.05d±1.44	2.56±0.62	14.35b	50.15c±3.07	1.92±0.61	12.03a	47.01b±1.38	4.21±0.60
*Trans*-3-Gg	5.75a	26.25ab±0.84	-0.92±0.12	6.63a	25.92a±1.39	0.90±0.79	6.55a	27.45b±2.29	-1.13±0.32	6.37a	29.84c±1.54	-1.27±0.25
*Trans*-2-G	1.37c	7.78c±0.95	-2.61±0.41	0.50a	2.46a±0.53	-2.21±1.65	0.55a	2.87a±0.88	-2.32±0.59	1.01b	5.89b±1.77	-4.50±0.31
*Cis*-4-GG	3.44b	9.33b±0.48	1.97±0.43	2.06a	5.74a±0.75	1.41±1.27	4.51c	9.63b±3.37	1.32±0.21	2.57ab	7.16a±1.25	2.83±0.49
*Cis*-3-Gg	1.93b	6.26c±0.94	-0.75±0.49	0.89a	2.08a±0.28	2.14±0.78	1.46a	4.35b±1.46	-1.26±0.50	1.16ab	3.78b±0.72	-0.91±0.45

() Number of samples analyzed for each country. * Δ mean content % between 0 and 24 h. Different letters between rows indicate significant differences at 0.05% level.

## 3. Experimental

### 3.1. Samples

A standard saffron sample belonging to commercial category I according to ISO 3632 (2003) [21] was used as control for all trials. Moreover, 46 samples of saffron (9 from Greece, 11 from Italy, 12 from Iran and 14 from Spain) were collected for studying the stability according to the production countries. 

### 3.2. Chemicals and reagents

a) Standards: Methyl 4-hydroxybenzoate (methyl paraben, MP, Sigma reference) and *n*-propyl 4-hydroxybenzoate (*n*-propyl paraben, PP, 99%) were purchased from Sigma-Aldrich (Madrid, Spain). Exact masses (1.00 g) of the chemical standards were dissolved first in acetonitrile and then water was added (50%) in a volumetric flask (500 mL). Different volumes of these stocking solutions (2 g/L) were added to the saffron aqueous extracts to obtain the following concentrations of parabens 50-100-150-200 mg/L.

b) Solvents: acetonitrile was purchased from Panreac (Barcelona, Spain) while water was purified through a Milli-Q System (Millipore, Bedford, MA, USA).

### 3.3. Procedure and Instrumentation

**Figure 3:**
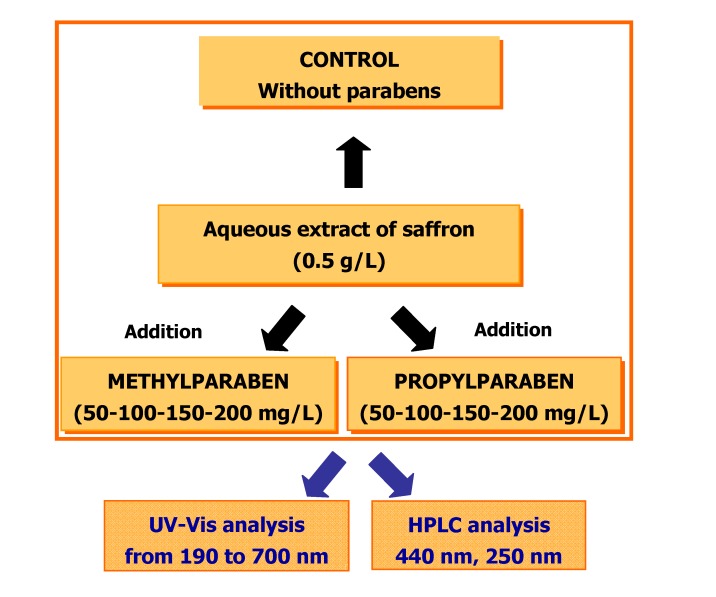
Scheme of workplan.

The saffron aqueous extract (500 mg/L) was prepared according to ISO 3632 (2003) [21]. Different volumes of a methyl paraben solution, placed in an amber vial, were then added to this saffron aqueous extract to obtain different concentrations (50-100-150-200 mg/L) of this paraben. For propyl paraben, the same procedure was followed. As control sample a saffron aqueous extract with no addition of parabens was used (Figure 3). The control sample was analysed every 40 min for 24 hours. The solution was homogenized and 20 μL was injected into an Agilent 1100 HPLC chromatograph (Palo Alto, CA) equipped with a 150 mm x 4.6 mm I.d., 5 μm Phenomenex (Le Pecq Cedex, France) Luna C_18_ column thermostated at 30 ºC. The solvents were water (A) and acetonitrile (B) using the following gradient: 80% A for 5 min to 20 % A in 15 min, at a flow rate of 0.8 mL/min. The DAD detector was set at 250 nm for the determination of picrocrocin, and at 440 nm for determining the crocetin esters. The 46 saffron samples were also analysed. For each, a saffron aqueous extract (500 mg/L) was prepared according to ISO 3632 (2003) [21]. This solution was then placed in an amber vial and 200 mg/L of propylparaben added. All experiments were carried out in triplicate at 25 ± 2 ºC. Each compound was identified by HPLC-DAD and the results were in agreement with literature [1,9]. Respective maxima in the UV–Vis region and retention times were used as means of identification. Due to the lack of pure standards for each crocetin ester, quantification was based on the equation:

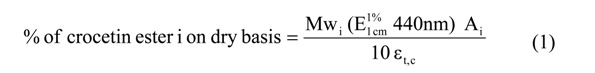

where Mwi stands for the molecular weight of the crocetin ester *i,* 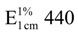 is the colouring strength, *A_i_* is the percentage peak area of the crocetin ester *i* at 440 nm, and *Є*_t,c_ is the molecular coefficient absorbance value (89000 for *trans*-crocetin esters and 63350 for *cis*-crocetin esters) [23].

### 3.4. Geographical sample differentiation according to the stability

47 saffron aqueous extract samples (control sample and 46 saffrons coming from different countries) were analysed by UV-Vis using a Perkin-Elmer Lambda 25 spectrophotometer (Norwalk, CT) at 257, 330 and 440 nm. 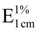 at 257 nm, 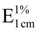 at 330 nm and 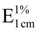 at 440 nm values were calculated according to ISO 3632 (2003) [21]. Every sample was measured in triplicate. The kinetic parameters of each reaction-reaction order, rate constants (*k*), and half-life periods (t_1/2_) were obtained using the integral method [24]. This method uses a trial-and-error procedure to find reaction order. If the order assumed is correct, the appropriate plot of the concentration-time data[concentration against time (zero-order), ln concentration against time (first-order), and concentration^-1^ against time (second-order)] should be linear. The result showing the best correlation coefficient (*R*^2^) was selected. 

### 3.5. Statistical analysis

Evaluation of the statistical significance of differences was performed using analysis of variance (ANOVA) with the aid of the SPSS 15.0 for Windows (SPSS Inc.) statistical program.

## 4. Conclusions

Regarding the control sample, all crocetin esters followed a first-order kinetic model confirming the data reported in the literature. The 3-Gg crocetin esters were more stable than the 4-GG ones for both *trans* and *cis*-isomers. A stabilizing effect towards crocetin esters was observed for both parabens, especially for propyl paraben. The saffron samples analysed with the addition of 200 mg/L of propyl paraben showed significant differences in the total crocetin ester content, except for Italian and Spanish samples. Unlike the remaining crocetin esters, significant differences in the content of *trans*-3Gg between countries were not found. No significant differences have been found with pricrocrocin, as it is a very stable molecule. Therefore, the addition of these compounds would permit the faster and cost-effective determination of crocetin esters of numerous samples using the autosampler HPLC systems, with the subsequent improvement of routine saffron analysis. 

## References

[1] Carmona M., Zalacain A., Sánchez A.M., Novella J.L., Alonso G.L. (2006). Crocetin Esters, Picrocrocin and its Related Compounds Present in *Crocus sativus* Stigmas and *Gardenia jasminoides* Fruits. Tentative Identification of Seven New Compounds by LC-ESI-MS. J. Agric. Food Chem..

[2] Carmona M., Zalacain Aramburu A., Pardo J.E., López E., Alvarruiz A., Alonso Díaz-Marta G.L. (2005). Influence of Different Drying and Aging Conditions on Saffron Constituents. J. Agric. Food Chem..

[3] Alonso G.L., Varón R., Gómez R., Navarro F., Salinas M.R. (1990). Auto-Oxidation in Saffron at 40ºC and 75% Relative Humidity. J. Food Sci..

[4] Alonso G.L., Sánchez M.A., Salinas M.R., Esteban-Infantes F.J. Inhibición de la autooxidación de las substancias responsables de las características del azafrán. Proceeding of the II Congreso Internacional de Química de la ANQUE.

[5] Alonso G.L., Varón R., Salinas M.R., Navarro F. (1993). Autooxidation of crocin and picrocrocin in saffron under different storage conditions. Boll.Chim. Farm..

[6] Alonso G.L., Sánchez M.A., Salinas M.R., Esteban-Infantes F.J. Cinética de la pérdida de poder colorante en extractos acuosos de azafrán a distintas temperaturas. Presented at the IX Congreso Nacional de Química.

[7] Tsimidou M., Tsatsaroni E. (1993). Stability of Saffron Pigments in Aqueous Extracts. J. Food Sci..

[8] Orfanou O., Tsimidou M., Charalambous G. (1995). Influence of Selected Additives on the Stability of Saffron Pigments in Aqueous Extracts. Food flavours: Generation, Analysis and Process Influence.

[9] Sánchez A.M., Carmona M., Ordoudi S., Tsimidou M., Alonso G.L. (2008). Kinetics of Individual Crocetin Ester Degradation in Aqueous Extracts of Saffron (*Crocus*
*sativus*L.) upon Thermal Treatment in the Dark. J. Agric. Food Chem..

[10] Carmona M., Sánchez A.M., Ferreres F., Zalacain A., Tomás-Barberán F., Alonso G.L. (2007). Identification of the flavonoid fraction in saffron spice by LC/DAD/MS/MS: Comparative Study of Samples from Different Geographical Origins. Food Chem..

[11] Castellar M.R. (1992). Biotransformación de la picrocrocin con β-glucosidasa inmovilizada. Doctoral thesis.

[12] Soni M.G., Carabin I.G., Burdock G.A. (2005). Safety Assessment of Esters of p-Hydroxybenzoic Acid (parabens). Food Chem. Toxicol..

[13] García-Jiménez J.F., Valencia M.C., Capitán-Vallvey L.F. (2007). Simultaneous Determination of Antioxidants, Preservatives and Sweetener Additives in Food and Cosmetics by Flow Injection Analysis Coupled to a Monolithic Column. Anal. Chim. Acta..

[14] Ali M.S., Chaudhary R.S., Takieddin M.A. (1999). Simultaneous Determination of Metronidazole Benzoate, Methylparaben, and Propylparaben by High-Performance Liquid Chromatograph. Drug Develop. Ind. Pharm..

[15] Mahuzier P.E., Altria K.D., Clark B.J. (1927). Selective and Quantitative Analysis of 4-Hydroxybenzoate Preservatives by Microemulsion Electrokinetic Chromatography. J. Chromatogr..

[16] Wang S.P., Chang C.L. (1998). Determination of Parabens in Cosmetic Products by Supercritical Fluid Extraction And Capillary Zone Electrophoresis. Anal. Chim. Acta.

[17] Kreuz D.M., Howard A.L., Ip D. (1999). Determination of Indinavir, Potassium Sorbate, Methylparaben, and Propylparaben in Aqueous Pediatric Suspensions. J. Pharm. Biomed.Anal..

[18] Lin Y.H., Chou S.S., Sheu F., Shyu Y.T. (2000). Simultaneous Determination of Sweeteners and Preservatives in Preserved Fruits by Micellar Electrokinetic Capillary Chromatography. J. Chromatogr. Sci..

[19] Labat L., Kummer E., Dallet P., Dubost J.P. (2000). Comparison of High-Performance Liquid Chromatography and Capillary Zone Electrophoresis for the Determination of Parabens in a Cosmetic Product. J. Pharm. Biomed. Anal..

[20] Skaria C.V., Gaisford S., O’Neill M.A.A., Buckton G., Beezer A.E. (2005). Stability Assessment of Pharmaceuticals by Isothermal Calorimetry: two Component Systems. Int. J. Pharm..

[21] (2003). ISO 3632-1,2 Technical Specification. Saffron (Crocus sativus L.). Part 1 (Specification) and Part 2 (Test methods)..

[22] Morteza P.H., Reza F.M., Nasrin S., Ehsan N., Ali R.S., Amini M. (2007). Deterioration of Parabens in Preserved Magnesium Hydroxide Oral Suspensions. J. Appl. Sci..

[23] Speranza G., Dadà G., Manitto P., Monti D., Gramatica P. (1984). 13-*cis*-crocin: a New Crocinoid of Saffron. Gazz.Chim. Ital..

[24] Fogler H.S., Amundson (1992). Elements of Chemical Reaction Engineering.

